# Comparative Analysis of Intravenous Opioids Versus Thoracic Epidural Anesthesia in Fractured Rib Pain Management: A Systematic Review and Meta-Analysis

**DOI:** 10.7759/cureus.51740

**Published:** 2024-01-06

**Authors:** Eslam Hussein Mohamed, Amr Elmoheen, Khalid Bashir, Mohamed Fayed, Mohammed Abdurabu, Mohammed Gafar Abdelrahim, Ali Elkandow, Kaleem Basharat, Stuart Lloyd, Ghassan Alwahsh, Hany A Zaki

**Affiliations:** 1 Emergency Medicine, Hamad Medical Corporation, Doha, QAT; 2 Emergency Medicine, Qatar University College of Medicine, Doha, QAT; 3 Emergency Medicine, Hamad Medical Corporation, Al Khor, QAT

**Keywords:** thoracic trauma, rib fractures, pain management, fentanyl, morphine, intravenous opioids, "epidural anesthesia", thoracic epidural analgesia

## Abstract

Rib fractures, common among trauma victims, lead to significant morbidity and mortality. Managing the associated pain is challenging, with IV opioids and thoracic epidural analgesia (TEA) being utilized. While epidural analgesia is often preferred for fractured rib pain, existing data encompasses both lumbar and thoracic approaches. This review aimed to compare TEA and IV opioids for persistent rib fracture pain. A comprehensive search across five databases yielded 987 articles, of which seven met the eligibility criteria. Outcomes were categorized into primary (pain reduction) and secondary (mortality, hospital/ICU stays, analgesia-related complications) endpoints. Analyzed with Review Manager (RevMan) Version 5.4.1 (2020; The Cochrane Collaboration, London, United Kingdom), the pooled data from two sources showed TEA significantly more effective in reducing pain than IV opioids (standardized mean difference* (*SMD): 2.23; 95%CI: 1.65-2.82; p < 0.00001). Similarly, TEA was associated with shorter ICU stays (SMD: 0.73; 95%CI: 0.33-1.13; p = 0.0004), while hospitalization duration showed no substantial difference (SMD: 0.82; 95%CI: -0.34-1.98). Mortality rates also did not significantly differ between TEA and IV opioids (risk ratio (RR): 1.20; 95%CI: 0.36-4.01; p = 0.77). Subgroup analysis revealed fewer pneumonia cases with TEA (RR: 2.06; 95%CI: 1.07-3.96; P = 0.03), with no notable disparities in other complications. While TEA's superiority in pain relief for rib fractures suggests it is the preferred analgesic, the recommendation's strength is tempered by the low methodological quality of supporting articles.

## Introduction and background

Rib fractures are frequent injuries in trauma patients, with a prevalence of roughly 10-15% [1-3]. Research suggests that 75% of these injuries originate from blunt thoracic trauma, primarily due to automobile accidents, whereas 25% are repercussions of penetrating injuries [4]. Fractured ribs have a mortality rate as high as 33% and substantial morbidity that results in patients needing frequent transfer to the intensive care unit (ICU) [5]. These premature deaths and morbidity are primarily caused by hypoventilation owing to pain, poor gas exchange attributed to injured lungs, and disrupted breathing mechanics. Moreover, research has demonstrated a correlation between age and severity of injuries with morbidity and death. Holcomb and colleagues established that four or more rib fractures were related to increased mortality, with an average death rate of 29% documented for patients with seven or more fractures [6]. On the other hand, Nirula and colleagues observed a death rate of 33% as a result of flail chest alone [7].

The pain brought about by rib fractures is challenging to alleviate; nevertheless, appropriate analgesia might rapidly avert hypoventilation and permit prolonged respiration, sufficient coughing with clearance of respiratory secretions, and adherence to chest physiotherapy. Moreover, adequate analgesia minimizes the frequency of respiratory complications and the need for breathing assistance. Therefore, analgesia for individuals with rib fractures must be quickly initiated in the emergency department (ED), not only to control pain and bring comfort but also for the sake of avoiding complications that arise during the next few days.

In previous years, IV opioids were viewed as the primary pain management therapy for rib fractures; however, adverse reactions such as respiratory impairment, delirium, and depressed cough reflex led to the adoption of various pain alleviating modalities, including thoracic epidural analgesia (TEA), intercostal blocks, intrapleural blocks, and paravertebral blocks. Recent guidelines have suggested the use of epidural analgesics over non-regional anesthesia for the treatment of rib fracture pain [8,9]. However, the data offered in these recommendations is of poor quality and generalizes epidural analgesia. Therefore, this meta-analysis was conducted to precisely evaluate the analgesic efficacy of TEA over IV opioids in patients with rib fractures.

## Review

Methodology

Literature Search

Two reviewers independently performed a systematic and structured literature search on five electronic databases, i.e., PubMed, MEDLINE (Medical Literature Analysis and Retrieval System Online), Embase, CENTRAL (Cochrane Central Register of Controlled Trials), and Google Scholar. The search involved identifying articles published up to May 2023, using the following keywords and Medical Subject Headings (MeSH) terms: (Thoracic epidural analgesia OR epidural anesthesia) AND (Intravenous opioids OR IV morphine OR IV fentanyl) AND (pain management OR pain control) AND (rib fractures OR traumatic rib injuries OR multiple rib fractures OR thoracic injuries OR chest injuries OR thoracic trauma). The reviewers also eliminated all close or exact duplicates and grey literature that would have undermined the scientific purpose of this research article.

Eligibility Criteria

An experienced reviewer was assigned to evaluate eligible studies, focusing on those that met specific criteria for inclusion in the analysis and review. These criteria encompassed human randomized trials or observational studies published in English, specifically those comparing the effectiveness of thoracic epidural analgesia with intravenous opioid analgesia in patients with rib fractures. Additionally, the studies needed to report outcomes such as pain scores, the duration of hospital stays, complications associated with analgesia, the length of intensive care unit stays, or mortality rates.

The reviewer also established specific exclusion criteria for the study selection process. This included excluding studies that involved animal subjects or were presented as conference abstracts, systematic reviews and meta-analyses, case reports, ongoing trials, and letters to the editor. Additionally, studies that employed lumbar epidural analgesia or intramuscular opioids were excluded. Furthermore, studies that directly compared the analgesic modalities being examined, but exclusively in patients who underwent surgical procedures, were also not considered for inclusion in the review.

Data Extraction and Definitions

Two independent reviewers retrieved data to carry out this review and recorded them in duplicate tables. The data collected included author ID (first author’s surname and year of publication), study design, participants’ characteristics, IV opioid and epidural anesthesia dosages, and outcomes of each study. Our analyses categorized the outcomes reported in the included studies as primary or secondary outcomes. The primary outcome was a reduction in pain, while secondary outcomes included length of hospital stay, ICU stay, ventilator duration, and complications related to the analgesia. In the event of discrepancies in the collected data, the two reviewers engaged in constructive discussions or consulted a third reviewer who helped to harmonize the data.

Pain reduction was defined using two different medical scales. The first scale was the verbal rating scale, which ranges from 0 to 10, with 0 considered as “no pain” and 10 regarded as “the worst imaginable pain” [10]. The other scale used was the 10-point visual analog scale, which designates a score of 0, “no pain,” and 10, “worst pain” [11].

Quality Assessment

The methodological quality of articles included in the present review was independently appraised with the Newcastle Ottawa Scale. This approach employs three aspects, i.e., selection, comparability, and outcomes, to measure quality. Four assessment questions were employed under the selection domain, while two assessment questions were used in the comparison domain. In addition, three assessment questions were to evaluate the quality of the research under the outcome domain. Once every article was reviewed using the appraisal criteria, the overall quality was assessed by translating the scores to the Agency of Healthcare Research and Quality (AHRQ) standards. According to these standards, the articles were graded as good, fair, or poor. Sound quality was assigned when the selection criterion was rated either 3 or 4, comparability was 1 or 2, and the result domain was rated 2 or 3. Moreover, fair quality was assigned for rating scores of 2 in the selection domain, 1 or 2 in the comparability domain, and 2 or 3 in the result domain. In contrast, poor quality scored 0 or 1 in the selection domain, 0 in the comparability domain, and 0 or 1 in the outcome domains.

Data Synthesis

The overall effect sizes for primary and secondary outcomes were calculated using the Review Manager (RevMan) Version 5.4.1 (2020; The Cochrane Collaboration, London, United Kingdom). Outcomes related to pain reduction, length of hospitalization, and ICU stay were continuous; therefore, calculations were performed using the standard mean difference (SMD). On the other hand, outcomes related to complications and mortality were binary; therefore, meta-analyses were performed using the risk ratio (RR). Moreover, the DerSimonian & Laird random effects model was used to estimate all effect sizes because it can counter the anticipated heterogeneity and provide conservative estimates. The heterogeneity was calculated using the I2 statistics, of which values greater than 50% indicated significant heterogeneity.

Results

Study Selection

**Figure 1:**
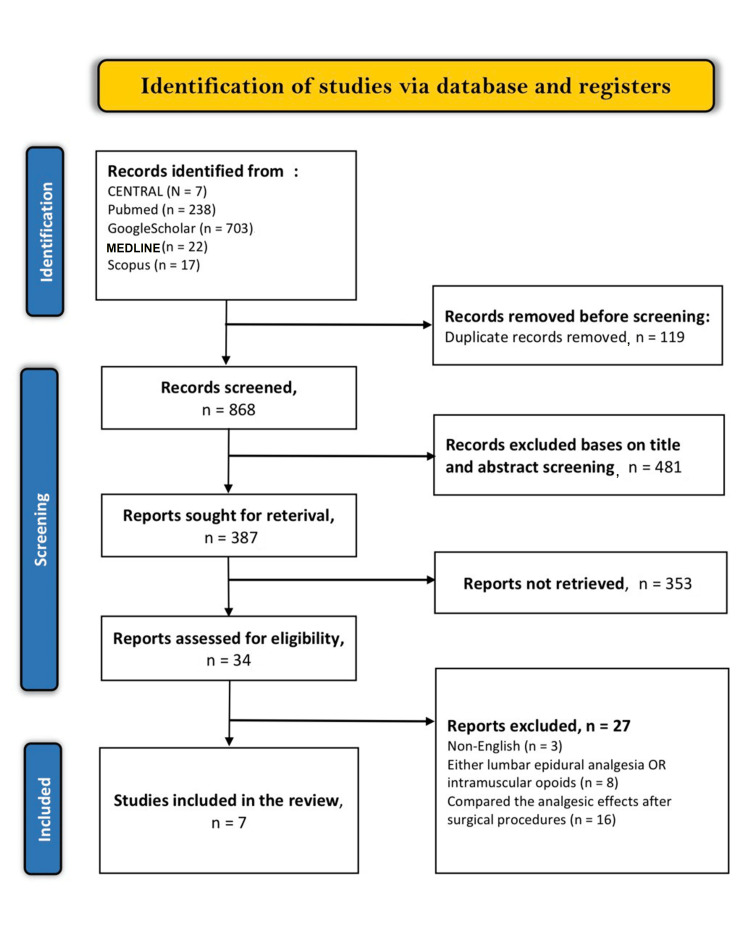
PRISMA flow diagram of the literature search results. PRISMA: Preferred Reporting Items for Systematic Reviews and Meta-Analyses

The present systematic review and meta-analysis adhere strictly to the Preferred Reporting Items for Systematic Reviews and Meta-Analyses (PRISMA) guidelines. Our systematic literature search on the aforementioned electronic databases resulted in 987 articles. These articles were analyzed for duplicates, of which 119 close or exact duplicates were excluded from the review. Afterward, the reviewers carried out a profound screening criterion on the titles and abstracts of the remaining articles and excluded 481 articles that did not meet the requirements. Out of the remaining 387 articles, the reviewers did not retrieve 353 articles because they were either ongoing trials, systematic reviews, case reports, case series, or letters to the editor. Finally, only seven articles met the inclusion criteria, while the other 27 articles were excluded: three were published in languages other than English, eight used lumbar epidural analgesia or intramuscular opioids, and 16 compared analgesic effects after surgical procedures. The entire selection criterion is shown in Figure 1.

Characteristics of the Included Studies

The summary of the characteristics and significant features of the seven included studies is shown in Table 1.

**Table 1:** Summary of the characteristics of the included studies. RCT: Randomized controlled trial; NR: Not reported; TEA: Thoracic epidural analgesia; IV: Intravenous; ICU: Intensive care unit; PCA: Patient controlled analgesia; ARDS: Acute respiratory distress syndrome; AIS: Abbreviated injury severity

Author ID	Study Design	Participants’ characteristics	IV opioid dosage	Thoracic epidural dosages	Outcomes
Waqar et al.,2013 [12]	Retrospective study	85 patients (64 males and 21 females; mean age: 47 (23–71) years).	NR	NR	Patients receiving TEA had significantly shorter analgesia days than those receiving IV opioids (4.25+1.2 vs. 5.5+3.2; p = 0.015). The duration spent in the ICU and hospital was shorter when receiving TEA as opposed to IV opioids (12+2.4 vs. 14+3.5 days; p = 0.002, and 19+3.1 vs 21+4.1; p = 0.01, respectively). No considerable difference was recorded in mortality, complication, pneumonia, and cardiac complications (p = 0.84, 0.86, 0.87, and 0.84, respectively).
Omar et al., 2018 [13]	Observational cohort study	30 patients (Ages: 18–65 years)	Loading dose of 0.1 mg/kg IV morphine followed by PCA of 1 mg/ml IV morphine in a bolus of 2 mg with a 10-minute lockout duration.	Continuous infusion of 20–25 mcg fentanyl in bupivacaine 0.125%.	24 hours after the administration of analgesia, patients who received TEA had significant pain reduction during coughing compared to those who received IV opioids (7.4±1.88 to 2.2±0.944 vs. 7.2±2.07 to 4.26±0.88, respectively). The duration of analgesia was similar for patients receiving TEA and IV opioids (8±1.08 vs 8.2±0.77, respectively). Patients who received TEA were less likely to develop pneumonia than their IV opioid counterparts (6.6% vs. 13.33%), atelectasis (13.33% vs. 26.44%), sepsis (6.66% vs. 13.33%), intubation (6.66%vs. 20%) hypotension (13.33% vs. 0%), and deaths (6.66% vs. 20%).
Bulger et al., 2004 [14]	A prospective RCT	46 patients (33 males and 13 females)	IV morphine, hydromorphone and fentanyl	Bupivacaine, morphine, and fentanyl were administered epidurally.	The duration spent in hospital and ICU was similar for patients receiving TEA and IV opioids (18±16 vs. 16±13, and 10±15 vs. 12±26 days, respectively). The incidence of pneumonia, ARDS, and mortality were similar between the TEA group and the IV opioids group (p = 0.15, 0.15, and 0.50, respectively).
Kieninger et al., 2005 [15]	Retrospective study	170 patients (72 males and 115 females; mean age: 77 (56–99) years)	NR	NR	Patients receiving TEA tend to spend more time in hospital than those who receive IV opioids (8.6±4.6 5.6±5.1 days; p = 0.0065). The frequency of pulmonary complications was higher in the TEA group with less injury severity (AIS<=2) than those in the IV opioids group (12/14 vs. 31/81; p = 0.001).
Moon et al., 1999 [16]	RCT	24 patients (14 male and 10 females)	Loading dose of 0.1 mg/kg IV morphine followed by PCA of 1 mg/ml IV morphine in a bolus of 2 mg with a 10-minute lockout duration.	Continuous infusion of bupivacaine 0.25% and morphine (0.005%) was initiated at a rate of 4-6 ml/hr using an infusion pump	24 hours after analgesia, patients receiving TEA had a significant pain reduction compared to IV opioids (5.8 vs. 7.4; p<0.05). Patients receiving TEA spent shorter time in ICU as opposed to IV opioids (3.8 vs. 4.1).
Ahmed et al., 2015 [17]	Prospective RCT	20 patients (15 males and 5 females; Ages: 18 – 55 years).	2 µg/kg of Iv fentanyl.	4 mL of 0.125% bupivacaine bolus followed by infusion of 4 mL/h with 2 µg/mL fentanyl as adjuvant.	ICU stay was shorter when using TEA as opposed to IV fentanyl (9.5±1.6 vs. 12.8±2.8 days; p = 0.004). The incidence of pneumonia, ARDS, and mortality did not differ between the IV opioids and TEA groups (p = 0.63, 0.35, and 1.00, respectively).
Bhaskar et al., 2023 [18]	Prospective observational study	46 patients (39 males and 7 females)	Continuous infusion of IV fentanyl at a rate of 2 µg/kg/h	Bupivacaine 0.125% with 2 μg/mL of fentanyl	Patients receiving TEA were admitted to the ICU for a shorter duration than those receiving IV fentanyl (10.17±4.697 vs. 13.83±4.96 days, respectively). TEA had a significant pain reduction than IV fentanyl at all measured intervals (p <0.001, at 6 hours, 12 hours, 24 hours, 36 hours, and 72 hours).

Quality Assessment Results

The quality appraisal shows that only one study had good methodological quality, while five had fair methodological quality, and one was of poor quality (Table 2).

**Table 2:** Methodological quality using Newcastle-Ottawa Scale

Study	Selection (/4)	Comparability (/2)	Outcome (/3)	Overall quality
Waqar et al., 2013 [12]	2	2	2	Fair
Omar et al., 2018 [13]	2	2	2	Fair
Bulger et al., 2004 [14]	3	2	2	Good
Kieninger et al., 2005 [15]	2	2	2	Fair
Moon et al., 1999 [16]	3	2	1	Poor
Ahmed et al., 2015 [17]	2	2	2	Fair
Bhaskar et al., 2023 [18]	2	2	2	Fair

The study with poor quality had some patients failing to complete it, which might have influenced their results. Moreover, none of the studies could score the maximum rating on patient selection because all the studies were carried out in single centers, which might not be a true representative of rib fracture patients.

Primary Outcome: Pain reduction

**Figure 2:**
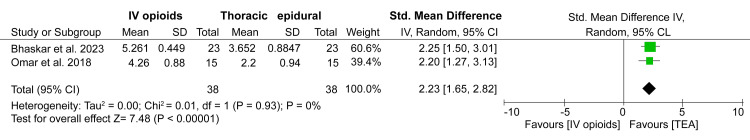
A forest plot comparing pain reduction between IV opioids and TEA groups. References: [13,18] TEA: Thoracic epidural analgesia

Only two studies included in our review reported changes in pain during the first 24 hours of receiving analgesia. The data pooled from these studies showed that patients receiving TEA had a significant pain reduction compared to those receiving IV opioids (SMD: 2.23; 95%CI: 1.65-2.82; p < 0.00001) (Figure 2).

Secondary Outcomes

**Figure 3:**
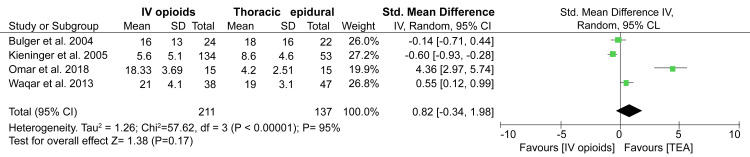
A forest plot comparing length of hospital stay between IV opioids and TEA groups. References: [12-15] TEA: Thoracic epidural analgesia

Length of hospital stay: The duration spent in hospitals after administering analgesia was reported in four studies, including 348 patients with fractured ribs. The pooled data from these studies demonstrated no significant difference in the length of hospitalization between patients who received IV opioids and those who received TEA (SMD: 0.82; 95%CI: -0.34-1.98) (Figure 3). However, the heterogeneity between studies was very large (I2 = 95%).

**Figure 4:**
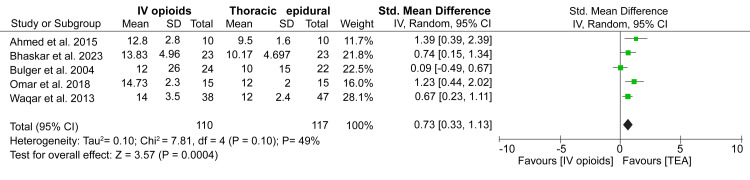
A forest plot comparing length of ICU stay between IV opioids and TEA groups. References: [12-14,17,18] TEA: Thoracic epidural analgesia

Length of ICU stay: Unlike the results on length of hospitalization, our meta-analysis showed a trend of longer ICU stays among patients who received IV opioids as opposed to those who received TEA (SMD: 0.73; 95%CI: 0.33-1.13; p = 0.0004) (Figure 4). Moreover, our statistical analysis has shown no significant heterogeneity between the studies (I2 = 49%).

Mortality

**Figure 5:**
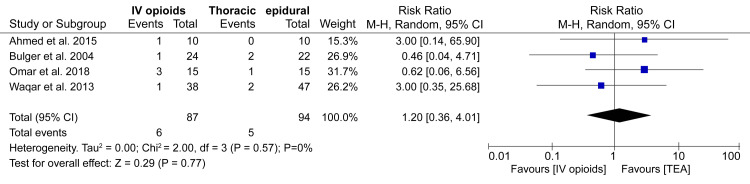
A forest plot comparing the incidence of mortality between IV opioids and TEA groups References: [12-14,17] TEA: Thoracic epidural analgesia

Rib fractures remain associated with significant morbidity and mortality. Therefore, it is essential to evaluate whether administering analgesia can reduce the mortality rate in patients with rib fractures. In our analysis of four studies, we found no considerable difference in the mortality risk between patients receiving IV opioids and TEA (RR: 1.20; 95%CI: 0.36-4.01; p = 0.77) (Figure 5).

Adverse Events

**Figure 6:**
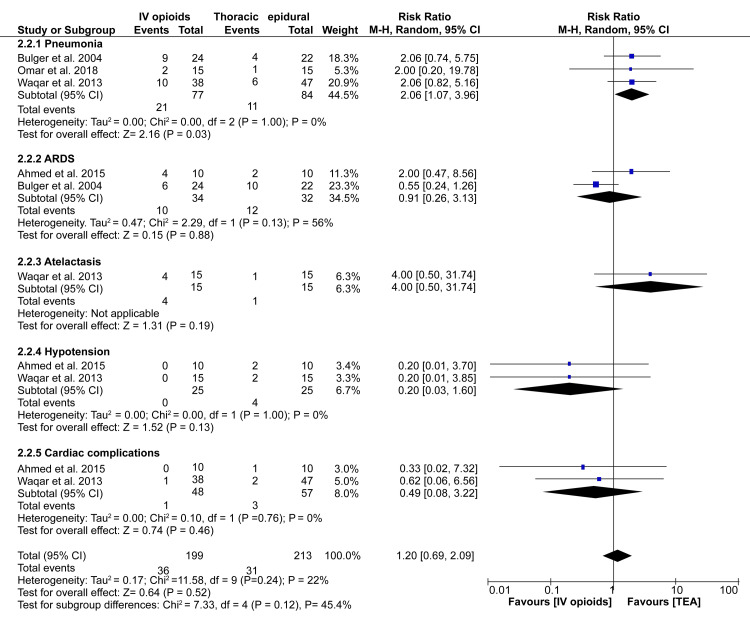
A forest plot showing subgroup analyses of adverse events between IV opioids and TEA groups. References: [12-14,17] TEA: Thoracic epidural analgesia

In our analysis, adverse effects associated with analgesia were categorized into pneumonia, acute respiratory distress syndrome (ARDS), atelectasis, hypotension, and cardiac complications. Pooled data from three sources show that patients treated with IV opioids were more likely to develop pneumonia than those who received TEA (RR: 2.06; 95%CI: 1.07-3.96; p = 0.03). However, our meta-analysis did not demonstrate any significant difference in the risk of ARDS (RR: 0.91; 95%CI: 0.26-3.13; p = 0.88), atelectasis (RR: 4.00; 95%CI: 0.50-31.74; p = 0.19), hypotension (RR: 0.20; 95%CI: 0.03-3.22; p =0.13) and cardiac complications (RR: 0.49; 95%CI: 0.08-3.22; p = 0.46) (Figure 6).

Discussion

Rib fractures continue to cause significant morbidity and mortality among trauma patients. The pain associated with these injuries contributes to voluntary splinting and muscle spasms, impairing respiratory function. Therefore, adequate pain management is essential for the therapy of rib fractures. Various analgesic modalities have been used for pain management; however, our meta-analysis mainly focused on the efficacy of IV opioids compared to TEA. Our results have shown that TEA significantly reduces pain and the time spent in the ICU compared to IV opioids. Moreover, patients managed with TEA seem to have lower incidences of pneumonia.

Our results on pain reduction are further reinforced by Moon and colleagues, who reported that patients receiving TEA had a significant reduction in pain during coughing than patients who received IV opioids [16]. Similarly, a recent meta-analysis of various analgesic modalities in patients with traumatic rib fractures concluded that epidural analgesia offers better pain relief than intravenous analgesia [19]. However, it is worth noting that this review had some methodological differences from ours. First, the review analyzed data for both lumbar and thoracic epidural analgesia. Second, the studies included in that review on epidural versus intravenous analgesia were fewer than ours. Finally, that review could not pool data for pain relief and made their conclusions directly from the outcomes of various studies. Previous research articles have also shown that TEA offers better pain relief than patient-controlled IV opioids post-thoracostomy [20,21]. With this evidence, it is logical that TEA provides better pain relief in patients with rib fractures.

Our meta-analysis has also shown that rib fracture patients receiving TEA tend to spend less time in the ICU than patients receiving IV opioids. This reduced ICU stay means that healthcare costs are also reduced because the length of stay significantly influences costs [22]. Contrary to our results, Bulger and colleagues found no considerable difference in the length of ICU stay between the TEA and IV opioids groups [14]. However, the results of that study should be interpreted with caution due to various methodological concerns. First, the trial had a small sample size, with only 37% of the patients meeting the eligibility criteria being included in the analysis. Second, the healthcare providers were not blinded to the analgesia provided, meaning they could have modified the analgesia to obtain better outcomes. For example, it is possible that the physicians were more aggressive in weaning ICU patients from mechanical ventilation, thereby influencing the ICU stay outcomes. Finally, the trial allowed a cross-over between treatment therapies. Therefore, the insignificant difference might have been due to TEA patients crossing over to the IV opioids group or vice versa.

Our findings have shown no considerable difference in the length of hospital stay between IV opioids and TEA groups. However, the heterogeneity in this outcome was high, meaning that the outcomes varied from study to study. For example, Omar and colleagues found that patients receiving TEA spent a significantly shorter time in the hospital than those receiving IV opioids [13]. On the other hand, Kieninger and colleagues found that IV opioids were associated with shorter hospital stays than TEA [15]. The authors of this study tried to evaluate the factors that influenced the length of hospital stay and found that patients with low injury severity scores (ISS) and those treated with TEA were more likely to spend more time in the hospital than those treated with IV opioids. However, no difference was recorded among patients with high ISS. Furthermore, the study found that the length of stay was longer for patients with comorbidities than those without any comorbidity. Despite the study favoring IV opioids over TEA, it cannot be used to provide guidelines for the use of IV opioids in patients with rib fractures due to various methodological limitations. First, the research was designed as a retrospective study, meaning that it accumulated all the limitations that come with this design. Secondly, selection bias was introduced to the patients treated with epidural analgesia. Finally, the fact that TEA was introduced in some patients due to deterioration on IV opioids may have biased the results.

When studying the efficacy of any intervention, it is also important to evaluate the side effects associated with the intervention before making any recommendations. Our analysis found that pneumonia frequency was significantly lower in the TEA group than with IV opioids. This low incidence among patients receiving TEA can be attributed to the fact that TEA allows for improved lung function [14,23]. Moreover, a previous meta-analysis comparing epidural analgesia to parenteral analgesia (intravenous or intramuscular) in patients with traumatic rib fractures mirrored our results by showing that patients receiving parenteral analgesia were more likely to develop pneumonia as opposed to those receiving epidural analgesia (p = 0.049) [24]. Apart from pneumonia, our analysis did not find any significant difference in other complications (i.e., ARDS, atelectasis, hypotension, and cardiac complications). However, hypotension was more common in patients receiving TEA than IV opioids. This was expected since epidural anesthesia can lead to blockage of the sympathetic nervous system, thus causing arterial and venous vasodilation [25].

Our meta-analysis suggests that TEA is superior to IV opioids in relieving pain associated with rib fractures; however, it is not without risks. First, it should be noted that TEA is an invasive pain control technique; therefore, complications such as bleeding are likely to occur. Currently, the risk of bleeding complications, especially after TEA, is unknown. However, a Swedish study recorded incidences of hematoma in eight patients receiving TEA and 17 receiving lumbar epidural analgesia [26]. Similarly, a prospective study in the United Kingdom recorded five bleeding events after TEA [27]. Second, TEA can cause neurological injuries. This is shown in the study by Bulger et al., where TEA had to be stopped in two patients due to deteriorating neurological status related to traumatic brain injury. Finally, TEA has been associated with an increased risk of accidental dural puncture, accidental intrathecal injection, and local anesthetic toxicity [21,28]. Therefore, physicians should weigh the benefit of improved pain control against the risks associated with thoracic epidural catheter placement when managing patients with rib fractures.

Moreover, the use of TEA in patients with rib fractures can be limited by various contraindications. A high proportion of rib fractures occurs due to high-energy blunt trauma, resulting in spinal injuries that interfere with epidural catheter placement or increased concern for developing neurological sequelae. In addition, patient refusal to undergo epidural anesthesia can limit the use of this analgesic modality. This is evident in the randomized trial by Bulger et al., where 80 of 408 eligible patients were excluded because they did not consent. Finally, TEA can be limited due to infection at the site of puncture, allergy to local anesthetic agents or opioids, coagulopathy, traumatic brain injury, and hemodynamic instability [29]. In these patients where TEA is contraindicated, IV analgesia with opioids supplemented with nonsteroidal anti-inflammatory drugs and paracetamol is the most preferred analgesic modality [17,18]. However, intramuscular, transdermal, or oral analgesia may also be used if feasible.

The pain associated with rib fractures may impair ventilation, resulting in the need for invasive mechanical ventilation (IMV). Furthermore, mispositioning of endotracheal or tracheostomy tubes during IMV is associated with a high risk of complications such as pneumoperitoneum, pneumothorax, and subcutaneous emphysema [30]. Therefore, it is essential to evaluate the role of analgesia in reducing the need for IMV and improving respiratory function. Waqar and colleagues showed that the intubation rates between the epidural and IV opioid groups were not significantly different (n=12 (25%) vs. n=9 (24%); p = 0.93) [12]. Conversely, Omar et al. found the incidence of intubation to be higher in the IV opioids group than in the TEA group (20% vs. 6.66%) [13]. However, the difference between the groups was statistically insignificant. Although TEA does not seem to reduce the need for IMV compared to IV opioids, evidence suggests that it is associated with a shorter duration of mechanical ventilation. After studying mechanically ventilated rib fracture patients, Ahmed and colleagues found that the duration of IMV was shorter for patients receiving TEA as opposed to IV opioids (p = 0.02) [17]. However, Bulger et al. did not find any considerable difference between TEA and IV opioid groups (p = 0.41) [14]. The insignificant difference in that study can be attributed to the fact that they allowed cross-over between the analgesic modalities.

Evidence also suggests that TEA can improve the respiratory function of rib fracture patients. According to Moon and colleagues, patients receiving TEA had a continuous increase (23%) in maximal inspiratory force (MIF), while patients receiving IV opioids had a gradual decline (15%) [16]. Moreover, the authors reported that by the end of the third day, the TEA group had much improved MIF than the IV opioids group (p <0.05). The tidal volume of mechanically ventilated patients was also studied, and it was found that on day 1, the difference between the two groups was insignificant. However, over the three-day follow-up, it was noticed that the tidal volume in the IV opioids gradually fell (i.e., by 56% from day 1). In contrast, the volume increased continuously in the TEA group (i.e., 45% increase) until it became statistically more significant than that of the IV opioid group. Contrary to the outcomes of this study, other research articles have recorded significant differences in tidal volume as early as 24 hours [17,24].

Although our meta-analysis was only focused on comparing the analgesic efficacy of TEA to IV opioids, evidence suggests that various agents used in the TEA offer different outcomes. Research by Block and colleagues suggests that using local anesthetics alone does not provide better pain control than a combination of local anesthetics and opioids [31]. Furthermore, using local anesthetics alone in TEA is limited due to hypotension. However, for patients with obstructive sleep apnea or intolerable side effects related to opioids, then TEA using local anesthetics alone can be preferred. Similarly, evidence shows that TEA using opioids alone does not improve postoperative pain control compared to parenteral opioids [31]. With this evidence, it is logical that even in patients with rib fractures, opioids alone in TEA may not provide more effective analgesia than IV opioids.

Moreover, the choice of opioids and local anesthetics to be used in TEA is very important. Research by Manion and colleagues shows that administration of bupivacaine 0.125% for TEA can reduce the incidences of nausea/vomiting, pruritus, and respiratory depression. However, this local anesthetic can result in increased incidences of hypotension and motor blockade. Moreover, this research article claims that the use of bupivacaine 0.1% or 0.05% together with hydromorphone 5-10 ug/ml or fentanyl 2-5 ug/ml can reduce the hemodynamic and side effects of opioids without causing any complications [32].

TEA also seems as effective as other regional anesthesia in reducing pain associated with rib fractures. A randomized trial comparing TEA to thoracic paravertebral infusion did not find any significant difference in pain reduction at rest (p = 0.426) or on coughing (p = 0.721) between the groups [23]. Similarly, a study comparing epidural analgesia to paravertebral analgesia among elderly patients with traumatic rib fractures did not find any difference in pain reduction between the groups, even after grouping the outcomes according to the number of fractures (p = 0.65, 0.78, and 0.29 for patients with two, three to five, and 6-12 rib fractures, respectively) [33]. However, there is evidence that TEA may provide more pain relief for rib fractures than intercostal blocks [34]. Furthermore, regional anesthesia seems more effective than parenteral opioids in patients with other injuries, such as hip fractures [35].

Limitations

The results presented in the current meta-analysis should be interpreted with caution due to several methodological limitations. First, our study disregarded data from studies published in other languages. Second, the studies used for meta-analyses are few and have low methodological quality, meaning that the quality of evidence from these studies was low and may influence our results and recommendations. Third, our meta-analysis did not group the TEA or IV opioids based on the agents used, meaning that it is difficult to recommend a particular agent to be used for TEA from our review. Fourth, considerable heterogeneity was recorded in the statistical analysis of the length of hospitalization. The varying sample sizes may have caused this heterogeneity, selection bias in some studies [15], and the allowance of cross-over between treatment groups [14]. Fifth, one of the studies included in this review evaluated pain reduction in rib fracture patients receiving TEA and IV opioids; however, due to poor reporting of the outcomes, it could not be used to calculate the overall effect size [16]. Finally, other complications related to analgesia, such as vomiting, respiratory depression, catheter-related injuries, itching, nausea, and rash, were not reported in our meta-analysis due to insufficient reporting. However, the most critical complications among rib fracture patients are pulmonary complications, and we sufficiently analyzed these in our study.

## Conclusions

Our study demonstrates that TEA offers superior pain reduction and decreases the duration of ICU stays when compared to IV opioids. Additionally, TEA appears to lower the incidence of pneumonia relative to IV opioids. Based on these findings, we advocate for the use of TEA as the preferred analgesic approach for patients with rib fractures. However, it is essential to note that the studies underpinning these recommendations are of low quality, underscoring the need for further high-quality randomized trials to solidify our results. While TEA provides enhanced pain management for rib fractures, its use is not without challenges, including various catheter-related complications that can impact its administration.

Consequently, it is crucial for physicians to carefully assess the risks and benefits of TEA in patients with rib fractures before proceeding with this treatment approach. In addition, future research should also focus on optimizing the application of TEA to minimize these complications and developing comprehensive guidelines to assist clinicians in making informed decisions about its use. This holistic approach will help patients receive the most effective and safe analgesic care.
